# The Safety and Immunogenicity of a Quadrivalent Influenza Subunit Vaccine in Healthy Children Aged 6–35 Months: A Randomized, Blinded and Positive-Controlled Phase III Clinical Trial

**DOI:** 10.3390/vaccines13050467

**Published:** 2025-04-26

**Authors:** Lili Huang, Guangfu Li, Yuhui Zhang, Xue Zhao, Kai Wang, Chunyu Jia, Wei Zhang, Jiebing Tan, Xiaofen Chen, Qin Li, Hongyan Jiang, Rui An, Wenna Leng, Yongli Yang, Youcai An, Yanxia Wang, Yaodong Zhang

**Affiliations:** 1 Henan Provincial Centre for Disease Control and Prevention, Zhengzhou 450016, China; 13643826177@163.com (L.H.); zwzzu@163.com (W.Z.); tanjiebingz@163.com (J.T.); 2 Ab&B Bio-Tech Co., Ltd. JS, Taizhou 225300, China; liguangfu@abbbio.com.cn (G.L.); zhangyuhui@abbbio.com.cn (Y.Z.); zhaoxue@abbbio.com.cn (X.Z.); wangkai@abbbio.com.cn (K.W.); jiachunyu@abbbio.com.cn (C.J.); chenxiaofen@abbbio.com.cn (X.C.); liqin@abbbio.com.cn (Q.L.); jianghongyan@abbbio.com.cn (H.J.); anrui@abbbio.com.cn (R.A.); lengwenna@abbbio.com (W.L.); anyoucai@abbbio.com.cn (Y.A.); 3 Department of Epidemiology and Public Health, College of Public Health, Zhengzhou University, Zhengzhou 450001, China; ylyang377@126.com

**Keywords:** influenza vaccine, safety, immunogenicity, subunit, clinical trial, children, phase III, reactogenicity, quadrivalent

## Abstract

**Background**: Influenza is a serious contagious disease caused by influenza virus. It is particularly dangerous for children, potentially leading to severe and even fatal complications. The aim of this study was to evaluate the safety and immunogenicity of two candidate quadrivalent influenza subunit vaccines in children aged 6–35 months. **Methods**: The subjects were randomly divided into three groups at a 1:1:1 ratio and received the corresponding vaccines: QIV-Sub-HD (Quadrivalent Influenza Subunit Vaccine, High Dose), QIV-Sub-LD (Quadrivalent Influenza Subunit Vaccine, Low Dose) and QIV-Split-LD (Quadrivalent Influenza Split-Virion Vaccine, Low Dose). Adverse events were recorded at 30 min, 0–7 days and 8–28 and 30 days after each dose of immunization. Serious adverse events (SAEs) were collected and reported within 6 months after the full vaccination. Blood samples were collected before the first dose and on 28 days, 3 months and 6 months after full vaccination for antibody detection to evaluate the immunogenicity and duration of immune responses. **Results**: The results showed that the relative and absolute criteria met the goals set by the clinical trial protocol, indicating that both vaccines are immunogenic. From the first dose to 30 days after full vaccination, the total incidence of adverse reactions in the QIV-Sub-HD, QIV-Sub-LD and QIV-Split-LD groups was 29.64%, 33.33% and 29.64%, respectively. The main symptoms were fever, cough, diarrhea and vomiting. No new safety concerns were identified. **Conclusions**: The quadrivalent influenza subunit vaccines candidate, manufactured by Ab&B Bio-tech Co., Ltd. JS., are safe and immunogenic in children aged 6–35 months.

## 1. Introduction

Influenza is a highly contagious viral infection that causes serious complications in children under 5 years of age and those with chronic illness and hospitalization, resulting in a significant public health threat and social burden [1,2,3]. Children can suffer from severe illness, such as severe pneumonia, necrotizing encephalitis, meningitis, etc. [4]. Currently, health authorities in many countries recommend annual influenza vaccination for children aged 6 months and older [5,6,7].

Influenza viruses are classified into types A, B, C and D based on the antigenicity of nucleoprotein and matrix protein [8]. Seasonal epidemics of influenza A and B viruses account for the majority of human respiratory disease burden. Although influenza A is historically considered as causing more severe disease, influenza B is also a major cause of epidemics in young children every few years, leading to illness and death [9,10]. Due to the high mutation rate and antigenic variation of influenza virus, the whole population is generally susceptible to influenza virus, but the highest infection rate and incidence were found in children during the epidemic season, with the vast majority of severe cases identified in healthy children ≤ 23 months or ≥10 years of age [11,12]. A systematic review and meta-analysis showed that the attack rate of influenza in children was 22.5% and that of symptomatic influenza was 12.7% [13]. The economic impact of childhood influenza is significant, including health care expenditures and indirect costs such as school absenteeism and parents’ loss of work productivity [14,15,16,17]. A national survey in China in 2013 showed that the economic burden of influenza in outpatient children under 5 years old was USD 196, of which the direct economic burden was USD 121; the cost of hospitalization for children under 5 years of age was USD 1508, of which the direct economic burden was USD 1295 [18].

Vaccination is the main approach to prevent influenza and its complications [19], but the vaccine effectiveness varies from year to year due to antigenic drift [20]. Based on the changes of the circulating influenza strains, the World Health Organization (WHO) recommends strains of virus to be used for annual influenza vaccine manufacturing to ensure that the vaccine matches the circulating strains [21].The traditional trivalent influenza vaccine (TIV) contains two influenza A and one influenza B virus strains, often resulting in a mismatch between the vaccine and the circulating strains [22,23] due to involvement of only one strain of the B lineage. In contrast to the TIV, the quadrivalent influenza vaccine (QIV) incorporates the Yamagata and Victoria B lineages in addition to influenza A strains (H1N1 and H3N2) to provide broader protection. TIV (split virion) and QIV (split virion) are the only two available influenza vaccines for children aged 6–35 months in China [6]. Compared to split virion vaccines, the influenza subunit vaccines manufactured by Ab&B Bio-tech Co., Ltd. JS, retain only the highly purified surface antigens, hemagglutinin and neuraminidase, while completely removing internal viral components such as the matrix protein and nucleoprotein, and therefore are likely to have better safety than split influenza virus vaccines [24,25] to meet the demand for quadrivalent influenza virus subunits in China.

Comprehensive phase I clinical trials have been conducted for the two different doses of the quadrivalent influenza subunit vaccine, and excellent safety and tolerability in children aged 6 to 35 months were demonstrated post-vaccination [26]. In terms of safety, the two dose groups (0.5 mL and 0.25 mL) exhibited similar and manageable adverse reaction rates. Within 28 days after the first dose, the total adverse reaction rates were 30.00% and 32.50% (*p* = 0.809), respectively. Within 30 days after the second dose, the rates further decreased to 12.50% and 20.51% (*p* = 0.337), indicating no increased safety risk with additional vaccination. All adverse reactions were predominantly systemic (e.g., fever), with no local reactions or Grade 3 or higher severe events reported. Regarding immunogenicity, the 0.5 mL dose group demonstrated significantly higher geometric mean titers (GMT) against H3N2, BV and BY subtypes compared to the 0.25 mL dose group (H3N2: 50.98 vs. 21.51, *p* = 0.032; BV: 48.40 vs. 23.57, *p* < 0.001; BY: 82.82 vs. 40.00, *p* = 0.006), while no significant difference was observed in H1N1 antibody GMT (127.73 vs. 77.13, *p* = 0.106). Antibody seroconversion rate analysis demonstrated that the 0.5 mL dose group had a significantly higher BV seroconversion rate compared to the 0.25 mL dose group (75.00% vs. 31.58%, *p* < 0.001). For other subtypes, although numerical increases were observed in the 0.5 mL group (H1N1: 85.00% vs. 76.32%, *p* = 0.331; H3N2: 50.00% vs. 31.58%, *p* = 0.098; BY: 75.00% vs. 57.89%, *p* = 0.109), these differences were not statistically significant. The ratio of antibody titer ≥ 1:40 data were consistent with the seroconversion results.

In this study, we conducted a Phase III, multicenter, randomized, blinded, positive-controlled and non-inferiority clinical trial in Henan Province to evaluate the safety and immunogenicity of the quadrivalent influenza subunit vaccine in healthy children aged 6 to 35 months.

## 2. Materials and Methods

### 2.1. Study Design

This phase III clinical trial took place at three locations in Henan Province, China, from 6 February 2023 to 26 February 2024 (NCT05645900). The aim of this study was to evaluate the safety and immunogenicity of two candidate quadrivalent influenza subunit vaccines in children aged 6–35 months.

In this study, 2772 subjects ranging from 6 to 35 months of age were included and randomly allocated to one of three groups, each receiving QIV-Sub-HD (Quadrivalent Influenza Subunit Vaccine, High Dose), QIV-Sub-LD (Quadrivalent Influenza Subunit Vaccine, Low Dose) or QIV-Split-LD (Quadrivalent Influenza Split-Virion Vaccine, Low Dose) in a 1:1:1 distribution. The study flow chart is shown in Figure S1. The sponsor provided qualified batches of investigational vaccine. This trial used a blinding method to ensure randomization and blinding. The randomization was conducted by a statistician using SAS 9.4 software developed by SAS Institute Inc. (Cary, NC, USA) and the block randomization method.

This study complies with the “Technical Guidelines for Clinical Trials of Vaccines” issued by the National Medical Products Administration (NMPA), the World Medical Association’s “Declaration of Helsinki” ethical guidelines for conducting medical research involving human participants, China’s current “Good Clinical Practice” (GCP) and all applicable regulations. Prior to the commencement of the study, the Medical Ethics Committee of the Henan Provincial Center for Disease Control and Prevention granted approval for the clinical trial protocol and the informed consent form. Researchers briefed the volunteers and their guardians (or delegates) about the informed consent form details for this clinical trial both orally and in writing. The informed consent form was jointly signed by all voluntary participants, their guardians (or delegates) and the study physicians.

### 2.2. Participants

Eligibility for study enrollment was determined based on fulfillment of the following criteria: (1) healthy infants and toddlers aged 6 to 35 months; (2) legal guardians voluntarily consenting for the subjects to participate in this study, and legal guardians/delegates signing the “Informed Consent Form” and being able to adhere to the clinical trial protocol requirements. Subjects were excluded before the first dose if they met any of the following conditions: (1) axillary temperature of 37.3 °C or higher at the time of enrollment; (2) laboratory-confirmed influenza virus infection within the past six months; (3) vaccination with any influenza vaccine (registered or experimental) within the previous 12 months or intended vaccination throughout the duration of the study; (4) allergy to any component of the study vaccine; (5) history of serious allergy reactions to any vaccine or medication; (6) infants aged 6 to 23 months who were premature (born before 37 weeks of gestation), had low birth weight (less than 2500 g), or had a history of challenging labor, asphyxia resuscitation or neurological impairment; (7) congenital malformations or developmental disorders, genetic defects, severe malnutrition, etc.; (8) presence of an acute illness, a severe chronic condition, or a flare-up of a chronic disease on vaccination day; (9) congenital or acquired immune deficiencies, lymphoma, leukemia or autoimmune diseases; (10) previously diagnosed with asthma, unstable and requiring emergency treatment, hospitalization, intubation or oral or intravenous corticosteroids in the past two years; (11) progressive neurological disorders, history of seizures, epilepsy, brain disease, Guillain-Barré syndrome, psychiatric history or family history; (12) severe cardiovascular disease; (13) absence of spleen, splenic dysfunction or splenectomy or other significant organ removal or partial removal; (14) prior diagnosis of coagulation disorders; (15) vaccination with a live attenuated vaccine within 14 days prior to vaccination or other vaccines within 7 days; (16) treatment with immunostimulants or immunosuppressants within the last 3 months (administered orally or via infusion for more than 14 consecutive days); (17) previous blood or blood product transfusions; (18) plans to move out of the area or long absences from the local area during the scheduled study visits before the study end date; (19) enrollment in other clinical trials is ongoing or planned in the near future; (20) any other condition deemed by the investigator as unsuitable for participation in this clinical trial. Subjects were excluded before the second dose if they met any of the following conditions: (1) serious allergic reaction; (2) severe adverse reactions caused by the first dose; (3) any individuals newly not fulfilling or fulfilling the pre-specified inclusion and exclusion criteria following vaccination underwent investigator-determined eligibility confirmation; (4) other investigator-determined exclusionary factors. 

### 2.3. Vaccines

QIV-Sub-HD and QIV-Sub-LD are manufactured by Ab&B Bio-tech Co., Ltd. JS (Taizhou, China). The QIV-Split-LD is manufactured by Hualan Biological Vaccine Co., Ltd. (Xinxiang, China). All of these vaccines are manufactured using the influenza A and B virus strains recommended by the World Health Organization (WHO) and do not contain preservatives or antibiotics. The specific virus strains included are A/Brisbane/02/2018 (H1N1)pdm09-like virus for H1N1, A/Kansas/14/2017 (H3N2)-like virus for H3N2, B/Phuket/3073/2013-like virus (B/Yamagata/16/88 lineage) for B(Y) and B/Colorado/06/2017-like virus (B/Victoria/2/87 lineage) for B(V).

QIV-Sub-HD is formulated as 0.5 mL per dose, with 15 μg of hemagglutinin for each influenza virus strain included. Both QIV-Sub-LD and the QIV-Split-LD are formulated as 0.25 mL per dose, with 7.5 μg of hemagglutinin for each influenza virus strain included. All of these vaccines are administered via intramuscular injection, with a total of 2 doses given 28 days apart.

### 2.4. Safety

Each subject was monitored for 30 min after immunization for immediate reaction and 0–7 days for active systemic safety after each dose of vaccine. From 7 days post-vaccination, adverse events were monitored with weekly follow-ups and participant self-reporting. Safety monitoring covered 0–28 or 0–30 days post each vaccination. Serious adverse events (SAEs) were tracked from initial vaccination until six months after completing the vaccination schedule.

### 2.5. Immunogenicity

Blood samples for influenza virus hemagglutination inhibition (HI) antibody testing were collected both pre-dose and at the 28-day follow-up after final dose administration. At the same time, in order to evaluate the immune persistence, blood samples were collected at 3 and 6 months after final dose administration, with HI antibody levels measured. The seroconversion rate (SCR), the ratio of antibody titer ≥ 1:40, GMT and geometric mean fold increase (GMFI) were calculated. Seroconversion was defined as HI antibody titer ≥ 1:40 post-immunization (<1:10 serum dilution) or a 4-fold rise in HI antibody titer post-immunization (≥1:10 serum dilution). SCR was defined as the rate of achieving seroconversion after immunization.

Immunogenicity was evaluated using the following criteria: Relative criteria: QIV-Sub-HD or QIV-Sub-LD should be non-inferior to the QIV-Split-LD after 28 days of the full vaccination. For the comparison of non-inferiority, both the GMT and SCR were used as the coprimary end points, that is, the lower limit of the 95% confidence interval (CI) of the difference in SCRs of four serotypes shared by the QIV-Sub and the QIV-Split should be ≥−10%. Additionally, the lower limit of the 95% CI of GMT_QIV-Sub_/GMT_QIV-Split_ should be ≥2/3 (equivalent to the difference on the log scale ≥ −0.176). The QIV-Sub can be considered non-inferior to the QIV-Split only if both conditions are met. Absolute criteria: For all four serotypes, the SCRs (two-sided 95% CI lower limit ≥ 30%) and the ratio of antibody titer ≥ 1:40 (two-sided 95% CI lower limit ≥ 60%) are met at 28 days after full vaccination of QIV-Sub-HD or QIV-Sub-LD in children aged 6–35 months.

Only when the above relative and absolute criteria are met can the robust vaccine immunogenicity in the 6–35-month cohort be determined.

### 2.6. Statistical Analysis

Statistical analysis was performed using SAS 9.4 software. In the non-inferiority test, the test level was α (one-sided) = 0.025. Statistical tests for all other conditions were descriptive or exploratory, and therefore no adjustment for multiplicity was applied; thus, the test level for other conditions was α = 0.05.

Safety analysis was performed by the *χ*^2^ test or Fisher’s exact test. The incidence of adverse events/reactions, grade 3 adverse events/reactions, and SAE rates were compared between the QIV-Sub-HD, QIV-Sub-LD and QIV-Split-LD groups.

The primary immunogenicity analysis was conducted using a three-step analysis strategy. Step 1, non-inferiority determination of the QIV-Sub-HD, was performed. The determination of Step 2 could proceed only if the non-inferiority determination of Step 1 was established. Step 2 was performed to determine the non-inferiority of the QIV-Sub-LD. Step 3 was used to determine the SCRs of 4 serotypes of HI antibody and whether the ratio of antibody titer was ≥1:40. If H0 was rejected in Step 1 but not in Step 2, only QIV-Sub-HD would be judged in Step 3. If H0 was rejected in both Steps 1 and 2, then Step 3 was judged for QIV-Sub-HD and QIV-Sub-LD, respectively.

The exploratory analysis was a comparison of antibodies after full vaccination between QIV-Sub-HD and QIV-Sub-LD groups. (1) The *χ*^2^ test, corrected *χ*^2^ test or Fisher’s exact test was used to compare the SCRs of 4 serotypes of HI antibody and the ratio of antibody titer ≥ 1:40 between QIV-Sub-HD and QIV-Sub-LD groups. (2) Two independent sample *t* tests were used to compare the difference in the GMTs of 4 serotypes of HI antibody between QIV-Sub-HD and QIV-Sub-LD groups. (3) The GMI (95%CI) between QIV-Sub-HD and QIV-Sub-LD groups was calculated.

The immune persistence analysis was conducted as follows: (1) Two independent sample t tests were used to compare the GMTs of 4 serotypes of HI antibody in QIV-Sub-HD, QIV-Sub-LD and QIV-Split-LD groups at 3 and 6 months after full vaccination. (2) The *χ*^2^ test, corrected *χ*^2^ test or Fisher’s exact test was used to compare the difference of SCR (95%CI) and the ratio of antibody titer ≥ 1:40 (95%CI) between QIV-Sub-HD, QIV-Sub-LD and QIV-Split-LD groups at 3 and 6 months after full vaccination.

## 3. Results

### 3.1. Subjects

**Figure 1:**
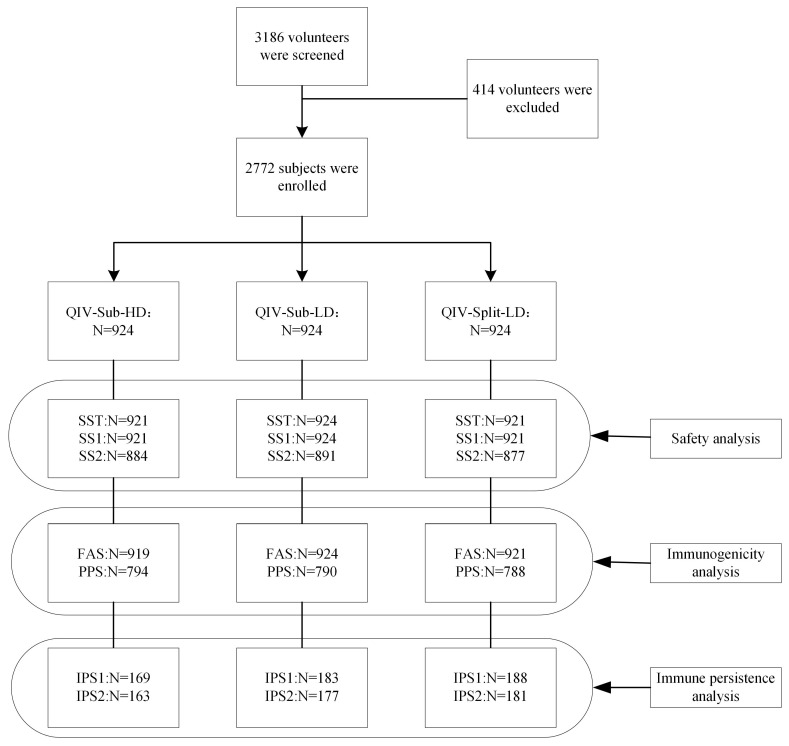
Flow chart of screening of all subjects. SST: Safety set total; SS1: Dose 1 safety set; SS2: Dose 2 safety set; FAS: Full analysis set; PPS: Per protocol set; IPS1: Immune persistence set 3 months post-vaccination; IPS2: Immune persistence set 6 months post-vaccination.

As shown in Figure 1, 3186 participants were screened, and 2772 subjects were enrolled, of whom 2766 completed the first vaccination and 2652 completed the last vaccination. During the trial, 40, 33 and 47 subjects did not complete the vaccination/safety observation in the QIV-Sub-HD group, QIV-Sub-LD group and QIV-Split-LD group, respectively. In addition, 52, 46 and 61 subjects did not complete the immunogenicity blood collection in the QIV-Sub-HD group, QIV-Sub-LD group and QIV-Split-LD group, respectively. Finally, 10, 3 and 5 visits were terminated due to adverse events in the QIV-Sub-HD, QIV-Sub-LD, and QIV-Split-LD groups, respectively.

The average age and gender composition of the subjects in the QIV-Sub-HD group, the QIV-Sub-LD group and the QIV-Split-LD group were balanced, with no statistically significant differences among the three groups (Table 1).

### 3.2. Safety

The total adverse reaction rates in QIV-Sub-HD, QIV-Sub-LD and QIV-Split-LD groups (from first vaccination through 30 days following the second vaccination) were 29.64%, 33.33% and 29.64%, respectively. Most adverse reactions occurred within the initial 7 days (including 30 min) post-vaccination, and there was no significant difference between any two groups. The solicited local adverse reaction rate in the QIV-Sub-HD group was significantly lower than that in QIV-Split-LD, and the unsolicited adverse reaction rate in QIV-Sub-LD was significantly higher than that in the QIV-Split HD group. There was no significant difference between any two groups in the other serotypes (Table 2).

From first vaccination through 30 days following the second vaccination., the adverse reactions in each group were mainly grade 1 or 2, and no grade 3 local adverse reactions occurred. The grade 1 adverse reaction rate in the QIV-Sub-HD group was significantly lower than that in QIV-Sub-LD and QIV-Split-LD. The vaccine-related grade 3 fever rate in the QIV-Sub-HD group was higher than that in QIV-Sub-LD and QIV-Split-LD, and the difference was statistically significant (Table 3).

From first vaccination through 30 days following the second vaccination, the most common adverse reactions were fever, runny nose, vomiting, cough, diarrhea and erythema at the injection site. Fever rates related to vaccination were 15.64% (13.35–18.14%), 14.94% (12.70–17.40%) and 11.83% (9.82–14.10%) in the QIV-Sub-HD, QIV-Sub-LD and QIV-Split-LD groups, respectively. The fever rate in the QIV-Sub-HD group was significantly higher than in QIV-Split-LD. The runny nose rates related to vaccination were 7.82% (6.17–9.74%), 10.39% (8.50–12.54%) and 7.27% (5.68–9.15%), respectively, with runny nose rate in the QIV-Sub-LD group being significantly higher than in QIV-Split-LD. The cough rates related to vaccination were 4.45% (3.21–5.99%), 5.30% (3.95–6.95%) and 5.43% (4.06–7.09%), respectively. The vomiting rates related to vaccination were 5.10% (3.77–6.73%), 5.41% (4.04–7.07%) and 4.99% (3.68–6.61%), respectively. The diarrhea rates related to vaccination were 3.91% (2.75–5.37%), 4.11% (2.93–5.60%) and 5.10% (3.77–6.73%), respectively. The erythema rates at the injection site related to vaccination were 2.28% (1.42–3.46%), 2.71% (1.76–3.97%) and 3.58% (2.48–5.00%), respectively (Table 4). There was only one vaccine-related SAE in the trial, which was diarrhea in a subject in the QIV-Sub-HD group. This SAE occurred on Day 11 after vaccination, which is relatively close to the time of vaccine administration, and diarrhea is one of the more common adverse reactions to the vaccine. Although the subject had received two antibiotics prior to hospital admission, and the diarrhea symptoms began on the third day of antibiotic use (suggesting a possible antibiotic-induced dysbiosis), we cannot completely rule out a potential association with the vaccine. Therefore, this SAE was considered possibly related to the vaccination.

### 3.3. Immunogenicity

PPS (Per Protocol Set) was used as the primary immunogenicity analysis. The non-inferiority test for the relative immunogenicity of QIV-Sub-HD and QIV-Sub-LD, as compared with QIV-Split-LD, was performed in a fixed sequence. In comparison with QIV-Split-LD, the lower limits of 95%CI of the SCRs of four serotypes in the QIV-Sub-HD and QIV-Sub-LD groups were all ≥−10%, and the lower limits of 95%CI of the GMT ratios were all ≥2/3, indicating that the both vaccines were non-inferior to the QIV-Split-LD (Table 5 and Table 6). When the absolute criteria were used, the lower limits of 95%CI of the SCR of QIV-Sub-HD and QIV-Sub-LD were >30%, and the lower limits of 95%CI of the ratio of antibody titer ≥ 1:40 were >60% in the total population (Table 6). In conclusion, the immunogenicity in subjects of QIV-Sub-HD and QIV-Sub-LD met both the relative and absolute goals set by the clinical trial protocol, indicating that the two vaccines elicit robust immunogenicity in the population aged 6–35 months.

In a comparative analysis of antibody responses 28 days after full vaccination, QIV-Sub-HD induced significantly higher SCRs for antibodies against H1N1, H3N2 and BV than QIV-Sub-LD, while the difference of the SCRs of the antibody against BY was not statistically significant (Table 6). Additionally, the GMT antibodies against H1N1 and H3N2 were significantly higher as induced by QIV-Sub-HD than QIV-Sub-LD. However, the differences in the GMT of antibodies against BV and BY were not statistically significant (Table 5). Moreover, the ratios of antibody titers ≥ 1:40 against H1N1, H3N2 and BV were statistically significantly higher as induced by QIV-Sub-HD than by QIV-Sub-LD, whereas the difference in the ratio of antibody titer ≥ 1:40 against BY was not statistically significant (Table 6).

At 3 and 6 months after full vaccination, there was a reduction in immune responses in all groups; however, the SCR, GMT and ratio of antibody titer ≥ 1:40 against H1N1 induced by QIV-Sub-HD were significantly higher than those induced by QIV-Sub-LD and QIV-Split-LD, suggesting that QIV-Sub-HD elicited a better long-lasting immunity than QIV-Sub-LD and QIV-Split-LD, while there were no significant differences in the SCR, GMT and ratio of antibody titer ≥ 1:40 among the other three serotypes (H1N1, H3N2 and BV) (Table 7 and Table 8).

Taking the above data into consideration, QIV-Sub-HD elicited better immunity in children aged 6–35 months, mainly reflected by induction of stronger responses against the H1N1, H3N2 and BV.

## 4. Discussion

In this phase III trial, we evaluated the safety and immunogenicity of two different doses of quadrivalent influenza subunit vaccine in children 6 to 35 months of age. This trial provided data to support the development of the quadrivalent influenza subunit vaccine for children 6–35 months.

The immunogenicity endpoints of this study included the SCR of HI antibody, the ratio of antibody titer ≥ 1:40, GMT and GMFI 28 days after the full immunization. The HI assay is a widely used and reproducible serological technique that is regarded as the gold standard for evaluating the immune capacity of influenza vaccines and licensure [27,28]. In this trial, after immunization with two doses of the quadrivalent influenza virus subunit vaccine, a significant humoral immune response was induced in the subjects, meeting both the relative and the absolute criteria. Compared to the antibody levels prior to vaccination, both QIV-Sub-HD and QIV-Sub-LD elicited a significant increase of GMTs of all four serotypes (H1N1, H3N2, BV and BY). At 28 days after full vaccination, the SCRs of antibodies against H1N1, H3N2, BV and BY were 98.74%, 84.01%, 93.32% and 82.87%, respectively, as induced by QIV-Sub-HD and 95.57%, 78.73%, 87.72% and 81.14%, respectively, as induced by QIV-Sub-LD. The results were consistent with similar studies [29,30]. In addition, except for serotype BY, QIV-Sub-HD was statistically higher than QIV-Sub-LD for the other three serotypes (H1N1, H3N2 and BV).

In this study, from immunization of the first dose to 30 days after the full immunization, the overall incidence rates of adverse reactions induced in the QIV-Sub-HD, QIV-Sub-LD, and QIV-Split-LD groups were 29.64%, 33.33% and 29.64%, respectively. In a previous Phase III clinical trial [31] evaluating the same vaccine (0.5 mL/dose), the overall adverse reaction rates within 0–30 days post-vaccination were 7.00% (4.70~9.96%) and 5.25% (3.28~7.91%) after immunization with QIV-Sub-HD and the QIV-Split-HD. The difference of the adverse reaction rates induced in two separate trials might be twofold in reason: Firstly, different ages of subjects were enrolled in the two trials, and adverse reactions were impacted by age. This trial was consistent with findings in China using similar products in the same age group [29,32] and had slightly lower adverse reaction data than the research results in the United States [30,33]. Secondly, this clinical trial was initiated shortly after the lifting of COVID-19 restrictions in the country (the first participant was enrolled and vaccinated on 6 February 2023 and the full immunization observation was completed 30 days after, on 13 May 2023), amid the prevalence of various infectious diseases. The adverse reactions occurring in the trial, i.e., fever, cough, diarrhea, vomiting and other symptoms, were considered to be related to the epidemic of coincidentally infectious diseases, such as norovirus infection and COVID-19, during the recruitment period. However, due to insufficient evidence to conclusively rule out vaccine-relatedness, and with strict adjudication of adverse event relatedness, the incidence of adverse reactions in each group was relatively high. In addition, regarding the significantly higher vaccine-related grade 3 fever rate in the QIV-Sub-HD group compared to the other two groups in this study, fever is one of the most common adverse reactions post-vaccination. Most cases were transient, resolving within 1 day. The between-group differences in the vaccine-related grade 3 fever rates may be attributed to coincidental infectious disease outbreaks (e.g., norovirus infection, COVID-19) during the subject enrollment period. However, due to insufficient evidence to definitively exclude a potential association with the vaccine, the causality of adverse events was adjudicated conservatively under maintained blinding. In general, the safety results of each group were similar, and the safety profile was good in healthy subjects aged 6–35 months. This clinical study had some limitations. Firstly, in terms of immunogenicity, only the humoral immune response was evaluated, not the cellular immune response. Secondly, the trial did not verify the protective efficacy of the vaccine.

## 5. Conclusions

QIV-Sub-HD and QIV-Sub-LD manufactured by Ab&b Bio-tech Co., Ltd. JS., demonstrated a good safety profile and immunogenicity when administered in a two-dose regimen to a healthy population aged 6–35 months. Comparative analysis of the safety results for the full course of QIV-Sub-HD and QIV-Sub-LD, as well as the immunogenicity results at 28 days, 3 months and 6 months after full vaccination, suggests that QIV-Sub-HD, while within acceptable safety parameters, shows relatively stronger immunogenicity results.

## Figures and Tables

**Table 1:** Baseline demographics and characteristics.

	QIV-Sub-HD Group	QIV-Sub-LD Group	QIV-Split-LD Group	*p*
**Age** (months)				
Medium (P25, P75)	22.73 (14.30, 29.00)	22.85 (14.52, 29.24)	23.67 (15.35, 29.97)	
Mean ± SD	21.86 ± 8.57	22.10 ± 8.64	22.72 ± 8.62	0.086
**Sex**				
Male (%)	481 (52.06)	455 (49.24)	454 (49.13)	0.363
Female (%)	443 (47.94)	469 (50.76)	470 (50.87)	

Note: Data are mean (SD), median (P25, P75) or numbers (%). The *χ*^2^ test or Fisher’s exact test was used to compare whether the sex composition of the subjects was balanced among the groups, One-way ANOVA was used to compare whether the mean age of the subjects was balanced among the groups.

**Table 2:** Adverse reaction rates by type (solicited/unsolicited) from first vaccination through 30 days following the second vaccination.

Type	Group	*n*	Adverse Reaction Rates (95%CI)	*p*
Total	QIV-Sub-HD	921	29.64 (26.71~32.71)	0.088 ^a^
	QIV-Sub-LD	924	33.33 (30.30~36.48)	0.088 ^b^
	QIV-Split-LD	921	29.64 (26.71~32.71)	>0.999 ^c^
Solicited	QIV-Sub-HD	921	24.86 (22.10~27.79)	0.200 ^a^
	QIV-Sub-LD	924	27.49 (24.63~30.49)	0.337 ^b^
	QIV-Split-LD	921	25.52 (22.73~28.46)	0.747 ^c^
Solicited local	QIV-Sub-HD	921	2.39 (1.50~3.59)	0.326 ^a^
	QIV-Sub-LD	924	3.14 (2.11~4.48)	0.140 ^b^
	QIV-Split-LD	921	4.45 (3.21~5.99)	0.015 ^c^
Solicited systemic	QIV-Sub-HD	921	23.34 (20.65~26.21)	0.377 ^a^
	QIV-Sub-LD	924	25.11 (22.34~28.03)	0.184 ^b^
	QIV-Split-LD	921	22.48 (19.82~25.31)	0.657 ^c^
Unsolicited	QIV-Sub-HD	921	8.69 (6.95~10.69)	0.066 ^a^
	QIV-Sub-LD	924	11.26 (9.29~13.47)	0.037 ^b^
	QIV-Split-LD	921	8.36 (6.65~10.34)	0.802 ^c^

Note: Data are rates (95%CI). The *χ*^2^ test or Fisher’s exact test was used to compare the adverse reaction rates among the groups. ^a^ QIV-Sub-HD vs. QIV-Sub-LD; ^b^ QIV-Sub-LD vs. QIV-Split-LD; ^c^ QIV-Sub-HD vs. QIV-Split-LD.

**Table 3:** The adverse reaction rate overview was graded according to symptoms from first vaccination through 30 days following the second vaccination.

Symptom	Group	*n*	Grade 1		*p*	Grade 2		*p*	Grade 3		*p*
			*n*	Rates (95%CI)		*n*	Rates (95%CI)		*n*	Rates (95%CI)	
**Total**	QIV-Sub-HD	921	130	14.12 (11.93~16.53)	0.004 ^a^	180	19.54 (17.03~22.25)	0.879 ^a^	15	1.63 (0.91~2.67)	0.140 ^a^
	QIV-Sub-LD	924	176	19.05 (16.56~21.73)	0.572 ^b^	178	19.26 (16.77~21.96)	0.122 ^b^	8	0.87 (0.37~1.70)	0.249 ^b^
	QIV-Split-LD	921	166	18.02 (15.59~20.66)	0.022 ^c^	152	16.50 (14.16~19.06)	0.090 ^c^	4	0.43 (0.12~1.11)	0.011 ^c^
**Systemic**											
Fever	QIV-Sub-HD	921	35	3.80 (2.66~5.25)	0.424 ^a^	100	10.86 (8.92~13.05)	0.687 ^a^	12	1.30 (0.68~2.26)	0.044 ^a^
	QIV-Sub-LD	924	42	4.55 (3.30~6.09)	0.068 ^b^	95	10.28 (8.40~12.42)	0.277 ^b^	4	0.43 (0.12~1.10)	>0.999 ^b^
	QIV-Split-LD	921	27	2.93 (1.94~4.24)	0.301 ^c^	81	8.79 (7.05~10.81)	0.137 ^c^	3	0.33 (0.07~0.95)	0.020 ^c^
Cough	QIV-Sub-HD	921	13	1.41 (0.75~2.40)	0.467 ^a^	28	3.04 (2.03~4.36)	0.701 ^a^	0	0.00 (0.00~0.40)	>0.999 ^a^
	QIV-Sub-LD	924	17	1.84 (1.08~2.93)	0.857 ^b^	31	3.35 (2.29~4.73)	0.789 ^b^	1	0.11 (0.00~0.60)	>0.999 ^b^
	QIV-Split-LD	921	18	1.95 (1.16~3.07)	0.365 ^c^	33	3.58 (2.48~5.00)	0.515 ^c^	0	0.00 (0.00~0.40)	-
Runny nose	QIV-Sub-HD	921	26	2.82 (1.85~4.11)	0.300 ^a^	47	5.10 (3.77~6.73)	0.082 ^a^	0	0.00 (0.00~0.40)	-
	QIV-Sub-LD	924	34	3.68 (2.56~5.10)	0.446 ^b^	65	7.03 (5.47~8.88)	0.009 ^b^	0	0.00 (0.00~0.40)	-
	QIV-Split-LD	921	28	3.04 (2.03~4.36)	0.782 ^c^	39	4.23 (3.03~5.74)	0.377 ^c^	0	0.00 (0.00~0.40)	-
Diarrhea	QIV-Sub-HD	921	27	2.93 (1.94~4.24)	0.516 ^a^	8	0.87 (0.38~1.70)	0.587 ^a^	1	0.11 (0.00~0.60)	>0.999 ^a^
	QIV-Sub-LD	924	32	3.46 (2.38~4.85)	0.329 ^b^	6	0.65 (0.24~1.41)	0.995 ^b^	1	0.11 (0.00~0.60)	>0.999 ^b^
	QIV-Split-LD	921	40	4.34 (3.12~5.87)	0.106 ^c^	6	0.65 (0.24~1.41)	0.592 ^c^	1	0.11 (0.00~0.60)	>0.999 ^c^
Vomiting	QIV-Sub-HD	921	19	2.06 (1.25~3.20)	0.187 ^a^	25	2.71 (1.76~3.98)	0.543 ^a^	3	0.33 (0.07~0.95)	0.997 ^a^
	QIV-Sub-LD	924	28	3.03 (2.02~4.35)	0.316 ^b^	21	2.27 (1.41~3.45)	0.643 ^b^	2	0.22 (0.03~0.78)	>0.999 ^b^
	QIV-Split-LD	921	21	2.28 (1.42~3.46)	0.749 ^c^	24	2.61 (1.68~3.85)	0.885 ^c^	1	0.11 (0.00~0.60)	0.617 ^c^
Constipation	QIV-Sub-HD	921	2	0.22 (0.03~0.78)	>0.999 ^a^	0	0.00 (0.00~0.40)	-	0	0.00 (0.00~0.40)	-
	QIV-Sub-LD	924	3	0.32 (0.07~0.95)	0.619 ^b^	0	0.00 (0.00~0.40)	-	0	0.00 (0.00~0.40)	-
	QIV-Split-LD	921	1	0.11 (0.00~0.60)	>0.999 ^c^	0	0.00 (0.00~0.40)	-	0	0.00 (0.00~0.40)	-
Nausea	QIV-Sub-HD	921	0	0.00 (0.00~0.40)	-	0	0.00 (0.00~0.40)	>0.999 ^a^	1	0.11 (0.00~0.60)	0.499 ^a^
	QIV-Sub-LD	924	0	0.00 (0.00~0.40)	0.249 ^b^	1	0.11 (0.00~0.60)	>0.999 ^b^	0	0.00 (0.00~0.40)	-
	QIV-Split-LD	921	2	0.22 (0.03~0.78)	0.479 ^c^	0	0.00 (0.00~0.40)	-	0	0.00 (0.00~0.40)	>0.999 ^c^
Headache	QIV-Sub-HD	921	0	0.00 (0.00~0.40)	-	1	0.11 (0.00~0.60)	0.499 ^a^	0	0.00 (0.00~0.40)	-
	QIV-Sub-LD	924	0	0.00 (0.00~0.40)	-	0	0.00 (0.00~0.40)	-	0	0.00 (0.00~0.40)	-
	QIV-Split-LD	921	0	0.00 (0.00~0.40)	-	0	0.00 (0.00~0.40)	>0.999 ^c^	0	0.00 (0.00~0.40)	-
Dyspnea	QIV-Sub-HD	921	0	0.00 (0.00~0.40)	-	0	0.00 (0.00~0.40)	>0.999 ^a^	0	0.00 (0.00~0.40)	-
	QIV-Sub-LD	924	0	0.00 (0.00~0.40)	-	1	0.11 (0.00~0.60)	>0.999 ^b^	0	0.00 (0.00~0.40)	-
	QIV-Split-LD	921	0	0.00 (0.00~0.40)	-	0	0.00 (0.00~0.40)	-	0	0.00 (0.00~0.40)	-
**Local**											
Erythema	QIV-Sub-HD	921	17	1.85 (1.08~2.94)	0.216 ^a^	4	0.43 (0.12~1.11)	0.132 ^a^	0	0.00 (0.00~0.40)	-
	QIV-Sub-LD	924	25	2.71 (1.76~3.97)	0.340 ^b^	0	0.00 (0.00~0.40)	0.499 ^b^	0	0.00 (0.00~0.40)	-
	QIV-Split-LD	921	32	3.47 (2.39~4.87)	0.030 ^c^	1	0.11 (0.00~0.60)	0.370 ^c^	0	0.00 (0.00~0.40)	-
Pain	QIV-Sub-HD	921	0	0.00 (0.00~0.40)	0.500 ^a^	0	0.00 (0.00~0.40)	-	0	0.00 (0.00~0.40)	-
	QIV-Sub-LD	924	2	0.22 (0.03~0.78)	>0.999 ^b^	0	0.00 (0.00~0.40)	-	0	0.00 (0.00~0.40)	-
	QIV-Split-LD	921	1	0.11 (0.00~0.60)	>0.999 ^c^	0	0.00 (0.00~0.40)	-	0	0.00 (0.00~0.40)	-
Induration	QIV-Sub-HD	921	0	0.00 (0.00~0.40)	-	0	0.00 (0.00~0.40)	>0.999 ^a^	0	0.00 (0.00~0.40)	-
	QIV-Sub-LD	924	0	0.00 (0.00~0.40)	0.249 ^b^	1	0.11 (0.00~0.60)	>0.999 ^b^	0	0.00 (0.00~0.40)	-
	QIV-Split-LD	921	2	0.22 (0.03~0.78)	0.479 ^c^	1	0.11 (0.00~0.60)	>0.999 ^c^	0	0.00 (0.00~0.40)	-
Swelling	QIV-Sub-HD	921	0	0.00 (0.00~0.40)	>0.999 ^a^	2	0.22 (0.03~0.78)	0.249 ^a^	0	0.00 (0.00~0.40)	-
	QIV-Sub-LD	924	1	0.11 (0.00~0.60)	0.369 ^b^	0	0.00 (0.00~0.40)	-	0	0.00 (0.00~0.40)	-
	QIV-Split-LD	921	4	0.43 (0.12~1.11)	0.133 ^c^	0	0.00 (0.00~0.40)	0.479 ^c^	0	0.00 (0.00~0.40)	-
Pruritus	QIV-Sub-HD	921	1	0.11 (0.00~0.60)	0.499 ^a^	0	0.00 (0.00~0.40)	-	0	0.00 (0.00~0.40)	-
	QIV-Sub-LD	924	0	0.00 (0.00~0.40)	-	0	0.00 (0.00~0.40)	0.499 ^b^	0	0.00 (0.00~0.40)	-
	QIV-Split-LD	921	0	0.00 (0.00~0.40)	>0.999 ^c^	1	0.11 (0.00~0.60)	>0.999 ^c^	0	0.00 (0.00~0.40)	-

Note: Data are numbers and rates. The *χ*^2^ test or Fisher’s exact test was used to compare the adverse reaction rates among the groups. ^a^ QIV-Sub-HD vs. QIV-Sub-LD; ^b^ QIV-Sub-LD vs. QIV-Split-LD; ^c^ QIV-Sub-HD vs. QIV-Split-LD.

**Table 4:** Symptomatic adverse reaction rate overview from first vaccination through 30 days following the second vaccination.

Symptom	Group	*n*	Adverse Reaction Rates (95%CI)	*p*
**Systemic**				
Fever	QIV-Sub-HD	921	15.64 (13.35~18.14)	0.676 ^a^
	QIV-Sub-LD	924	14.94 (12.70~17.40)	0.051 ^b^
	QIV-Split-LD	921	11.83 (9.82~14.10)	0.018 ^c^
Cough	QIV-Sub-HD	921	4.45 (3.21~5.99)	0.396 ^a^
	QIV-Sub-LD	924	5.30 (3.95~6.95)	0.905 ^b^
	QIV-Split-LD	921	5.43 (4.06~7.09)	0.333 ^c^
Runny nose	QIV-Sub-HD	921	7.82 (6.17~9.74)	0.055 ^a^
	QIV-Sub-LD	924	10.39 (8.50~12.54)	0.018 ^b^
	QIV-Split-LD	921	7.27 (5.68~9.15)	0.659 ^c^
Diarrhea	QIV-Sub-HD	921	3.91 (2.75~5.37)	0.824 ^a^
	QIV-Sub-LD	924	4.11 (2.93~5.60)	0.310 ^b^
	QIV-Split-LD	921	5.10 (3.77~6.73)	0.217 ^c^
Vomiting	QIV-Sub-HD	921	5.10 (3.77~6.73)	0.767 ^a^
	QIV-Sub-LD	924	5.41 (4.04~7.07)	0.687 ^b^
	QIV-Split-LD	921	4.99 (3.68~6.61)	0.915 ^c^
Constipation	QIV-Sub-HD	921	0.22 (0.03~0.78)	>0.999 ^a^
	QIV-Sub-LD	924	0.32 (0.07~0.95)	0.619 ^b^
	QIV-Split-LD	921	0.11 (0.00~0.60)	>0.999 ^c^
Nausea	QIV-Sub-HD	921	0.11 (0.00~0.60)	>0.999 ^a^
	QIV-Sub-LD	924	0.11 (0.00~0.60)	0.998 ^b^
	QIV-Split-LD	921	0.22 (0.03~0.78)	>0.999 ^c^
Headache	QIV-Sub-HD	921	0.11 (0.00~0.60)	0.499 ^a^
	QIV-Sub-LD	924	0.00 (0.00~0.40)	-
	QIV-Split-LD	921	0.00 (0.00~0.40)	>0.999 ^c^
Dyspnea	QIV-Sub-HD	921	0.00 (0.00~0.40)	>0.999 ^a^
	QIV-Sub-LD	924	0.11 (0.00~0.60)	>0.999 ^a^
	QIV-Split-LD	921	0.00 (0.00~0.40)	-
**Local**				
Erythema	QIV-Sub-HD	921	2.28 (1.42~3.46)	0.558 ^a^
	QIV-Sub-LD	924	2.71 (1.76~3.97)	0.280 ^b^
	QIV-Split-LD	921	3.58 (2.48~5.00)	0.097 ^c^
Pain	QIV-Sub-HD	921	0.00 (0.00~0.40)	0.500 ^a^
	QIV-Sub-LD	924	0.22 (0.03~0.78)	>0.999 ^b^
	QIV-Split-LD	921	0.11 (0.00~0.60)	>0.999 ^c^
Induration	QIV-Sub-HD	921	0.00 (0.00~0.40)	>0.999 ^a^
	QIV-Sub-LD	924	0.11 (0.00~0.60)	0.614 ^b^
	QIV-Split-LD	921	0.33 (0.07~0.95)	0.248 ^c^
Swelling	QIV-Sub-HD	921	0.22 (0.03~0.78)	0.998 ^a^
	QIV-Sub-LD	924	0.11 (0.00~0.60)	0.369 ^b^
	QIV-Split-LD	921	0.43 (0.12~1.11)	0.683 ^c^
Pruritus	QIV-Sub-HD	921	0.11 (0.00~0.60)	0.499 ^a^
	QIV-Sub-LD	924	0.00 (0.00~0.40)	0.499 ^b^
	QIV-Split-LD	921	0.11 (0.00~0.60)	>0.999 ^c^

Note: Data are rates (95%CI). The *χ*^2^ test or Fisher’s exact test was used to compare the adverse reaction rates among the groups. ^a^ QIV-Sub-HD vs. QIV-Sub-LD; ^b^ QIV-Sub-LD vs. QIV-Split-LD; ^c^ QIV-Sub-HD vs. QIV-Split-LD.

**Table 5:** Comparison of GMTs and GMFI of antibodies 28 days after full vaccination.

Stage of Vaccination	Strain	Group	*n*	GMT (95%CI)	*p*	GMT Ratio (95%CI)	GMFI (95%CI)
**Pre-vaccination**	**H1N1**	QIV-Sub-HD	794	5.10 (5.04~5.16)	0.232 ^a^	-	-
		QIV-Sub-LD	790	5.16 (5.08~5.24)	0.568 ^b^	-	-
		QIV-Split-LD	788	5.13 (5.05~5.20)	0.569 ^c^	-	-
	**H3N2**	QIV-Sub-HD	794	8.87 (8.24~9.54)	0.784 ^a^	-	-
		QIV-Sub-LD	790	8.74 (8.10~9.42)	0.026 ^b^	-	-
		QIV-Split-LD	788	7.80 (7.30~8.33)	0.011 ^c^	-	-
	**BV**	QIV-Sub-HD	794	8.35 (7.75~9.00)	0.439 ^a^	-	-
		QIV-Sub-LD	790	8.72 (8.06~9.44)	0.274 ^b^	-	-
		QIV-Split-LD	788	9.30 (8.56~10.10)	0.060 ^c^	-	-
	**BY**	QIV-Sub-HD	794	21.20 (20.12~22.35)	0.845 ^a^	-	-
		QIV-Sub-LD	790	21.36 (20.29~22.48)	0.265 ^b^	-	-
		QIV-Split-LD	788	22.27 (21.13~23.46)	0.195 ^c^	-	-
**Post-dose 2**	**H1N1**	QIV-Sub-HD	794	351.64 (327.63~377.41)	<0.001 ^a^	1.29 (1.16~1.44)	68.93 (64.17~74.04)
		QIV-Sub-LD	790	272.05 (250.77~295.14)	0.005 ^b^	1.18 (1.05~1.33)	52.72 (48.58~57.22)
		QIV-Split-LD	788	230.09 (211.35~250.48)	<0.001 ^c^	1.53 (1.37~1.71)	44.86 (41.18~48.86)
	**H3N2**	QIV-Sub-HD	794	110.60 (100.70~121.47)	0.005 ^a^	1.21 (1.06~1.38)	12.48 (11.61~13.41)
		QIV-Sub-LD	790	91.41 (83.16~100.49)	0.581 ^b^	1.04 (0.91~1.18)	10.46 (9.70~11.29)
		QIV-Split-LD	788	88.13 (80.59~96.37)	0.001 ^c^	1.25 (1.10~1.43)	11.30 (10.43~12.25)
	**BV**	QIV-Sub-HD	794	149.08 (135.22~164.36)	0.057 ^a^	1.15 (1.00~1.34)	17.84 (16.80~18.96)
		QIV-Sub-LD	790	129.16 (115.57~144.36)	0.498 ^b^	0.95 (0.82~1.10)	14.81 (13.78~15.92)
		QIV-Split-LD	788	136.09 (122.81~150.81)	0.207 ^c^	1.10 (0.95~1.26)	14.64 (13.67~15.67)
	**BY**	QIV-Sub-HD	794	172.47 (158.72~187.43)	0.566 ^a^	1.04 (0.92~1.17)	8.13 (7.52~8.80)
		QIV-Sub-LD	790	166.44 (152.27~181.94)	0.117 ^b^	1.11 (0.98~1.26)	7.79 (7.19~8.44)
		QIV-Split-LD	788	150.45 (137.52~164.58)	0.029 ^c^	1.15 (1.01~1.30)	6.76 (6.24~7.31)

Note: The two-sample independent *t*-test was used to compare the differences in antibody GMTs. ^a^ QIV-Sub-HD vs. QIV-Sub-LD; ^b^ QIV-Sub-LD vs. QIV-Split-LD; ^c^ QIV-Sub-HD vs. QIV-Split-LD.

**Table 6:** Comparison of ratio of antibody titer ≥ 1:40 and SCRs of antibodies 28 days after full vaccination.

Stage of Vaccination	Strain	Group	*n*	Ratio of Antibody Titer ≥ 1:40 (95%CI)	*p*	SCRs (95%CI)	SCR Rate Differences	*p*
**Pre-vaccination**	**H1N1**	QIV-Sub-HD	794	0.25 (0.03~0.91)	0.678 ^a^	-	-	-
		QIV-Sub-LD	790	0.51 (0.14~1.29)	0.685 ^b^	-	-	-
		QIV-Split-LD	788	0.25 (0.03~0.91)	>0.999 ^c^	-	-	-
	**H3N2**	QIV-Sub-HD	794	19.65 (16.94~22.58)	0.888 ^a^	-	-	-
		QIV-Sub-LD	790	19.37 (16.67~22.30)	0.035 ^b^	-	-	-
		QIV-Split-LD	788	15.36 (12.91~18.06)	0.025 ^c^	-	-	-
	**BV**	QIV-Sub-HD	794	15.74 (13.28~18.47)	0.700 ^a^	-	-	-
		QIV-Sub-LD	790	16.46 (13.94~19.23)	0.180 ^b^	-	-	-
		QIV-Split-LD	788	19.04 (16.35~21.95)	0.084 ^c^	-	-	-
	**BY**	QIV-Sub-HD	794	28.59 (25.47~31.87)	0.917 ^a^	-	-	-
		QIV-Sub-LD	790	28.35 (25.23~31.64)	0.160 ^b^	-	-	-
		QIV-Split-LD	788	31.60 (28.36~34.97)	0.192 ^c^	-	-	-
**Post-dose 2**	**H1N1**	QIV-Sub-HD	794	98.74 (97.70~99.39)	<0.001 ^a^	98.74 (97.70~99.39)	3.17 (1.56~4.94)	<0.001 ^a^
		QIV-Sub-LD	790	95.57 (93.89~96.89)	0.023 ^b^	95.57 (93.89~96.89)	2.68 (0.37~5.03)	0.023 ^b^
		QIV-Split-LD	788	92.89 (90.87~94.59)	<0.001 ^c^	92.89 (90.87~94.59)	5.85 (3.89~7.80)	<0.001 ^c^
	**H3N2**	QIV-Sub-HD	794	86.40 (83.82~88.71)	0.007 ^a^	84.01 (81.27~86.49)	5.27 (1.44~9.09)	0.007 ^a^
		QIV-Sub-LD	790	81.39 (78.50~84.05)	0.968 ^b^	78.73 (75.71~81.54)	1.32 (−2.76~5.40)	0.525 ^b^
		QIV-Split-LD	788	81.47 (78.58~84.13)	0.008 ^c^	77.41 (74.33~80.29)	6.59 (2.72~10.47)	0.001 ^c^
	**BV**	QIV-Sub-HD	794	93.45 (91.50~95.07)	<0.001 ^a^	93.32 (91.36~94.96)	5.60 (2.73~8.51)	<0.001 ^a^
		QIV-Sub-LD	790	87.85 (85.36~90.05)	0.831 ^b^	87.72 (85.23~89.93)	0.67 (−2.62~3.95)	0.690 ^b^
		QIV-Split-LD	788	88.20 (85.74~90.37)	<0.001 ^c^	87.06 (84.51~89.32)	6.27 (3.35~9.19)	<0.001 ^c^
	**BY**	QIV-Sub-HD	794	97.10 (95.69~98.16)	0.168 ^a^	82.87 (80.07~85.43)	1.73 (−2.05~5.52)	0.369 ^a^
		QIV-Sub-LD	790	95.82 (94.18~97.11)	0.992 ^b^	81.14 (78.23~83.81)	5.25 (1.20~9.29)	0.011 ^b^
		QIV-Split-LD	788	95.81 (94.17~97.10)	0.165 ^c^	75.89 (72.74~78.84)	6.98 (3.01~10.96)	0.001 ^c^

Note: The *χ*^2^ test, corrected *χ*^2^ test or Fisher’s exact test was used to compare the differences in the ratio of antibody titers ≥1:40 or SCRs. ^a^ QIV-Sub-HD vs. QIV-Sub-LD; ^b^ QIV-Sub-LD vs. QIV-Split-LD; ^c^ QIV-Sub-HD vs. QIV-Split-LD.

**Table 7:** Comparison of GMTs and GMFI of antibodies 3 months and 6 months after full vaccination.

Stage of Vaccination	Strain	Group	*n*	GMT (95%CI)	*p*	GMT Ratio (95%CI)	GMFI (95%CI)
**3 months Post-dose 2**	**H1N1**	QIV-Sub-HD	169	114.77 (97.96~134.46)	0.022 ^a^	1.32 (1.04~1.67)	22.58 (19.27~26.46)
		QIV-Sub-LD	183	86.95 (72.71~103.98)	0.743 ^b^	0.96 (0.75~1.23)	16.94 (14.15~20.27)
		QIV-Split-LD	188	90.68 (75.87~108.39)	0.052 ^c^	1.27 (1.00~1.61)	17.94 (15.01~21.44)
	**H3N2**	QIV-Sub-HD	169	43.96 (35.39~54.59)	0.153 ^a^	1.24 (0.92~1.67)	6.82 (5.75~8.09)
		QIV-Sub-LD	183	35.43 (28.92~43.41)	0.044 ^b^	0.74 (0.56~0.99)	5.48 (4.68~6.41)
		QIV-Split-LD	188	47.57 (38.82~58.29)	0.600 ^c^	0.92 (0.69~1.24)	6.68 (5.73~7.78)
	**BV**	QIV-Sub-HD	169	74.92 (61.42~91.38)	0.487 ^a^	1.11 (0.83~1.47)	10.49 (9.25~11.89)
		QIV-Sub-LD	183	67.72 (55.16~83.14)	0.871 ^b^	0.98 (0.74~1.29)	9.24 (8.12~10.51)
		QIV-Split-LD	188	69.29 (57.39~83.64)	0.573 ^c^	1.08 (0.82~1.42)	8.87 (7.88~9.99)
	**BY**	QIV-Sub-HD	169	72.20 (62.34~83.63)	0.203 ^a^	1.15 (0.93~1.43)	6.36 (5.34~7.57)
		QIV-Sub-LD	183	62.78 (53.61~73.51)	0.641 ^b^	1.05 (0.84~1.32)	5.19 (4.42~6.11)
		QIV-Split-LD	188	59.57 (50.94~69.65)	0.080 ^c^	1.21 (0.98~1.50)	4.81 (4.13~5.60)
**6 months Post-dose 2**	**H1N1**	QIV-Sub-HD	163	58.65 (49.68~69.24)	0.042 ^a^	1.30 (1.01~1.67)	11.58 (9.82~13.66)
		QIV-Sub-LD	177	45.16 (37.33~54.64)	0.838 ^b^	0.97 (0.74~1.27)	8.79 (7.27~10.63)
		QIV-Split-LD	181	46.44 (38.40~56.18)	0.069 ^c^	1.26 (0.98~1.62)	9.25 (7.65~11.19)
	**H3N2**	QIV-Sub-HD	163	26.93 (21.39~33.91)	0.113 ^a^	1.29 (0.94~1.78)	4.14 (3.46~4.95)
		QIV-Sub-LD	177	20.80 (16.63~26.01)	0.100 ^b^	0.77 (0.57~1.05)	3.21 (2.70~3.82)
		QIV-Split-LD	181	26.86 (21.80~33.09)	0.986 ^c^	1.00 (0.74~1.37)	3.76 (3.24~4.38)
	**BV**	QIV-Sub-HD	163	47.62 (38.50~58.90)	0.394 ^a^	1.14 (0.84~1.56)	6.58 (5.75~7.52)
		QIV-Sub-LD	177	41.60 (33.13~52.22)	0.772 ^b^	0.96 (0.70~1.30)	5.62 (4.83~6.55)
		QIV-Split-LD	181	43.52 (35.42~53.46)	0.549 ^c^	1.09 (0.81~1.47)	5.50 (4.88~6.19)
	**BY**	QIV-Sub-HD	163	52.29 (45.29~60.36)	0.709 ^a^	1.04 (0.84~1.29)	4.64 (3.92~5.49)
		QIV-Sub-LD	177	50.20 (42.82~58.85)	0.649 ^b^	1.05 (0.84~1.31)	4.16 (3.53~4.90)
		QIV-Split-LD	181	47.71 (40.92~55.62)	0.392 ^c^	1.10 (0.89~1.35)	3.83 (3.31~4.45)

Note: The two-sample independent *t*-test was used to compare the differences in antibody GMTs. ^a^ QIV-Sub-HD vs. QIV-Sub-LD; ^b^ QIV-Sub-LD vs. QIV-Split-LD; ^c^ QIV-Sub-HD vs. QIV-Split-LD.

**Table 8:** Comparison of ratio of antibody titer ≥ 1:40 and SCRs of antibodies 3 months and 6 months after full vaccination.

Stage of Vaccination	Strain	Group	*n*	The Ratio of Antibody Titer ≥ 1:40 (95%CI)	*p*	SCRs (95%CI)	SCR Rate Differences (95%CI)	*p*
**3 months Post-dose 2**	**H1N1**	QIV-Sub-HD	169	90.53 (85.08~94.49)	0.039 ^a^	90.53 (85.08~94.49)	8.02 (0.81~15.13)	0.029 ^a^
		QIV-Sub-LD	183	83.06 (76.83~88.19)	0.191 ^b^	82.51 (76.22~87.72)	4.85 (−3.31~12.92)	0.242 ^b^
		QIV-Split-LD	188	77.66 (71.03~83.40)	0.001 ^c^	77.66 (71.03~83.40)	12.87 (5.46~20.28)	0.001 ^c^
	**H3N2**	QIV-Sub-HD	169	59.17 (51.36~66.66)	0.657 ^a^	58.58 (50.76~66.09)	1.75 (−8.51~11.93)	0.740 ^a^
		QIV-Sub-LD	183	56.83 (49.32~64.12)	0.905 ^b^	56.83 (49.32~64.12)	0.45 (−9.55~10.43)	0.931 ^b^
		QIV-Split-LD	188	57.45 (50.04~64.61)	0.741 ^c^	56.38 (48.98~63.59)	2.20 (−8.07~12.46)	0.675 ^c^
	**BV**	QIV-Sub-HD	169	82.84 (76.29~88.20)	0.633 ^a^	82.84 (76.29~88.20)	1.97 (−6.18~9.98)	0.633 ^a^
		QIV-Sub-LD	183	80.87 (74.42~86.30)	0.314 ^b^	80.87 (74.42~86.30)	4.28 (−4.08~12.54)	0.314 ^b^
		QIV-Split-LD	188	76.60 (69.88~82.45)	0.144 ^c^	76.60 (69.88~82.45)	6.24 (−2.06~14.55)	0.144 ^c^
	**BY**	QIV-Sub-HD	169	92.31 (87.21~95.84)	0.478 ^a^	73.96 (66.67~80.40)	6.20 (−3.32~15.50)	0.201 ^a^
		QIV-Sub-LD	183	90.16 (84.90~94.07)	0.031 ^b^	67.76 (60.47~74.47)	6.06 (−3.65~15.59)	0.222 ^b^
		QIV-Split-LD	188	82.45 (76.24~87.60)	0.005 ^c^	61.70 (54.35~68.68)	12.26 (2.67~21.86)	0.014 ^c^
**6 months Post-dose 2**	**H1N1**	QIV-Sub-HD	163	77.30 (70.10~83.49)	0.017 ^a^	77.30 (70.10~83.49)	11.76 (2.13~21.03)	0.017 ^a^
		QIV-Sub-LD	177	65.54 (58.04~72.51)	0.350 ^b^	65.54 (58.04~72.51)	4.76 (−5.20~14.58)	0.350 ^b^
		QIV-Split-LD	181	60.77 (53.26~67.93)	0.001 ^c^	60.77 (53.26~67.93)	16.53 (6.94~26.12)	0.001 ^c^
	**H3N2**	QIV-Sub-HD	163	45.40 (37.60~53.37)	0.505 ^a^	44.79 (37.00~52.76)	4.11 (−6.35~14.46)	0.444 ^a^
		QIV-Sub-LD	177	41.81 (34.45~49.44)	0.696 ^b^	40.68 (33.37~48.30)	3.11 (−6.94~13.08)	0.547 ^b^
		QIV-Split-LD	181	39.78 (32.59~47.31)	0.292 ^c^	37.57 (30.49~45.06)	7.22 (−3.18~17.61)	0.174 ^c^
	**BV**	QIV-Sub-HD	163	61.35 (53.42~68.86)	0.422 ^a^	60.74 (52.79~68.28)	4.80 (−5.65~15.08)	0.370 ^a^
		QIV-Sub-LD	177	57.06 (49.42~64.46)	0.509 ^b^	55.93 (48.29~63.37)	3.45 (−6.82~13.60)	0.513 ^b^
		QIV-Split-LD	181	53.59 (46.04~61.02)	0.146 ^c^	52.49 (44.95~59.94)	8.25 (−2.20~18.70)	0.123 ^c^
	**BY**	QIV-Sub-HD	163	86.50 (80.28~91.34)	0.122 ^a^	59.51 (51.55~67.12)	2.45 (−7.98~12.77)	0.648 ^a^
		QIV-Sub-LD	177	80.23 (73.59~85.82)	0.589 ^b^	57.06 (49.42~64.46)	1.81 (−8.39~11.96)	0.730 ^b^
		QIV-Split-LD	181	77.90 (71.15~83.72)	0.038 ^c^	55.25 (47.69~62.63)	4.26 (−6.19~14.71)	0.425 ^c^

Note: The *χ*^2^ test, corrected *χ*^2^ test or Fisher’s exact test was used to compare the differences in the ratio of antibody titers ≥1:40 or SCRs ^a^ QIV-Sub-HD vs. QIV-Sub-LD; ^b^ QIV-Sub-LD vs. QIV-Split-LD; ^c^ QIV-Sub-HD vs. QIV-Split-LD.

## Data Availability

Data will be made available on request.

## Supplementary Materials

The following supporting information can be downloaded at: https://www.mdpi.com/article/10.3390/vaccines13050467/s1, Figure S1: Study Flow Chart.
