# Correction: Huang et al. The Safety and Immunogenicity of a Quadrivalent Influenza Subunit Vaccine in Healthy Children Aged 6–35 Months: A Randomized, Blinded and Positive-Controlled Phase III Clinical Trial. *Vaccines* 2025, *13,* 467

**DOI:** 10.3390/vaccines13080826

**Published:** 2025-08-01

**Authors:** Lili Huang, Guangfu Li, Yuhui Zhang, Xue Zhao, Kai Wang, Chunyu Jia, Wei Zhang, Jiebing Tan, Xiaofen Chen, Qin Li, Hongyan Jiang, Rui An, Wenna Leng, Yongli Yang, Youcai An, Yanxia Wang, Yaodong Zhang

**Affiliations:** 1 Henan Provincial Centre for Disease Control and Prevention, Zhengzhou 450016, China; 13643826177@163.com (L.H.); zwzzu@163.com (W.Z.); tanjiebingz@163.com (J.T.); 2 Ab&B Bio-Tech Co., Ltd. JS, Taizhou 225300, China; liguangfu@abbbio.com.cn (G.L.); zhangyuhui@abbbio.com.cn (Y.Z.); zhaoxue@abbbio.com.cn (X.Z.); wangkai@abbbio.com.cn (K.W.); jiachunyu@abbbio.com.cn (C.J.); chenxiaofen@abbbio.com.cn (X.C.); liqin@abbbio.com.cn (Q.L.); jianghongyan@abbbio.com.cn (H.J.); anrui@abbbio.com.cn (R.A.); lengwenna@abbbio.com (W.L.); anyoucai@abbbio.com.cn (Y.A.); 3 Department of Epidemiology and Public Health, College of Public Health, Zhengzhou University, Zhengzhou 450001, China; ylyang377@126.com

The authors would like to make the following corrections to this published paper [1]. 

First, the formulation of QIV-Split-LD was incorrectly described. All underlying data, analyses, and conclusions remain accurate. 

A correction has been made to Section 2. Methods, Sub-section 2.3 Vaccines, Paragraph 2:

“QIV-Sub-HD is formulated as 0.5 mL per dose, with 15 µg of hemagglutinin for each influenza virus strain included. Both QIV-Sub-LD and the QIV-Split-LD are formulated as 0.25 mL per dose, with 7.5 µg of hemagglutinin for each influenza virus strain included.”

Second, the original sentence incorrectly attributed the significantly higher runny nose rate to the QIV-Sub-HD group instead of the QIV-Sub-LD group, based on the presented data and statistical analysis (QIV-Sub-LD: 10.39% vs QIV-Split-LD: 7.82%), it is the QIV-Sub-LD group that demonstrated the significantly higher rate relative to QIV-Split-LD. This is solely a textual error; the underlying data, statistics, tables, and scientific conclusions are correct.

A correction has been made to Section 3. Results, Sub-section 3.2 Safety, Paragraph 3:

“The runny nose rates related to vaccination were 7.82% (6.17–9.74%), 10.39% (8.50–12.54%) and 7.27% (5.68–9.15%), respectively, with runny nose rate in the QIV-Sub-LD group being significantly higher than in QIV-Split-LD.”

The authors state that the scientific conclusions are unaffected. This correction was approved by the Academic Editor. The original publication has also been updated.
