# Supplementary material for: The Safety and Immunogenicity of a Quadrivalent Influenza Subunit Vaccine in Healthy Children Aged 6–35 Months: A Randomized, Blinded and Positive-Controlled Phase III Clinical Trial
Source: Vaccines (Basel). 2025 Apr 26;13(5):467. doi: 10.3390/vaccines13050467 (PMC12116156; doi:10.3390/vaccines13050467)
Supplement: Supplementary file 1 [file vaccines-13-00467-s001.zip › vaccines-3493076-supplementary.pdf]

Supplementary Method

Sample Size Estimation

The calculation of sample size must comply with the requirements of the *Drug Registration Management Measures* issued by the **Center for Drug Evaluation (CDE)** and meet statistical standards. Based on the *Technical Guidelines for Clinical Research of Seasonal Influenza Virus Vaccines (Draft for Comment)* issued by the **CDE** (November 2021) and the immunogenicity assumptions of this trial, the sample sizes for the primary immunization phase and the immunogenicity persistence phase are estimated separately.

(1) Sample Size Estimation for Immunogenicity Analysis

① **Non-Inferiority Test Based on Antibody Seroconversion Rate**:

Referencing the Phase III clinical trial results of the QIV-Split-LD in children aged 6–35 months, the seroconversion rate of HI antibodies for four strains at 28 days post-full vaccination ranged between 60% and 90%. This trial uses GMT and seroconversion rate as co-primary endpoints, and non-inferiority is established only if both endpoints meet non-inferiority criteria. A fixed sequential testing approach is used to evaluate the immunogenicity of the 0.5 mL/dose test vaccine and the 0.25 mL/dose test vaccine. Thus, no adjustment for Type I error is applied (α = 0.025, one-sided). The total power ($1\text{–}\beta$) is set to 0.9. To account for potential inflation of Type II error due to four shared serotypes, a power of $1\text{–}\beta$ = 0.975 is used for each comparison. The non-inferiority margin is set to Δ= −0.10. The control seroconversion rate is conservatively assumed as P_0=0.60, with a 1:1 allocation ratio between test and control groups. Using PASS 2020’s Non-Inferiority for the Difference Between Two Proportions module (Z-test with pooled variance), each group requires 738 subjects. Accounting for a 20% dropout rate, each group needs 923 subjects (=738/0.8). Considering blinding factors, 924 subjects per group are allocated.

② **Non-Inferiority Test Based on GMT**:

Based on Phase III trial results of the QIV-Split-LD, the standard deviation (SD) of HI antibody GMT at 28 days post-vaccination for four strains ranged between 0.7 and 0.8 (log scale). GMT and seroconversion rate are co-primary endpoints, and non-inferiority requires both to meet criteria. Using the same sequential testing approach (α = 0.025, one-sided; total $1\text{–}\beta$ = 0.9, adjusted to $1\text{–}\beta$ = 0.975 per comparison), the non-inferiority margin is set to Δ=−0.176 (log scale, equivalent to a post-vaccination GMT ratio of test/control ≥ 2/3). Assuming an SD of 0.8 and a 1:1 allocation ratio, PASS 2020’s Two-Sample T-Tests for Non-Inferiority Assuming Equal Variance module calculates 636 subjects per group. With a 20% dropout rate, each group requires 795 subjects (=636/0.8).

③ **Confidence Interval Estimation for Proportions**:

Using PASS 2020’s Confidence Intervals for a Proportion module (Clopper-Pearson exact method), with a 95% confidence level ($1-\alpha$=0.95), P=0.60, and precision =0.06(i.e., 10% of P), each group requires 271 subjects. Including a 20% dropout rate, each group needs 339 subjects.

The results of the above three sample size estimation were integrated. Following the principle of maximizing sample size and considering site allocation and blinding parameters, 924 subjects per group (QIV-Sub-HD, QIV-Sub-LD, and QIV-Split-LD groups) are allocated, and 2,772 subjects.

(2) Sample Size Estimation for Immunogenicity Persistence Analysis：

To estimate the ratio of antibody titer ≥ 1:40 at 6 months post-full vaccination, the Confidence Intervals for a Proportion module in PASS 2020 was employed. Using the Clopper-Pearson exact method for confidence interval estimation, with a confidence level $1-\alpha=0.95$, $P=0.5$(for the ratio of antibody titer ≥ 1:40 at 6 months), and precision=0.08, each of the QIV-Sub-HD group and QIV-Sub-LD group requires 160 subjects.

Therefore, 180 subjects per group(QIV-Sub-HD, QIV-Sub-LD, and QIV-Split-LD groups) were enrolled for immunogenicity persistence blood sampling at 3 months post-full vaccination, resulting in a total of 540 subjects.

Supplementary Figure S1 – Study Flow Chart

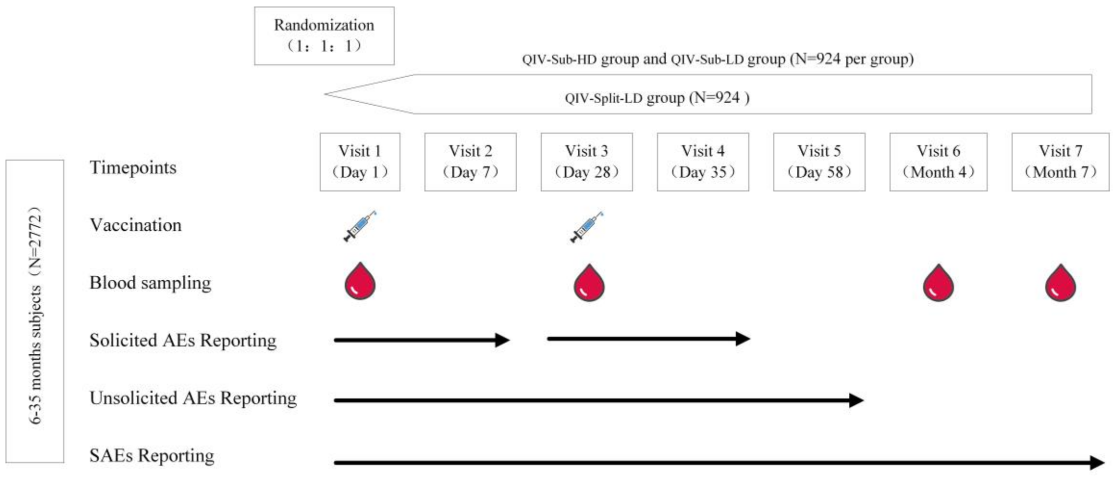

Note: Blood sampling collection at Visit 6 and Visit 7 was only available to immune persistence study subjects. QIV-Sub-HD: Quadrivalent Influenza Subunit Vaccine, High Dose. QIV-Sub-LD: Quadrivalent Influenza Subunit Vaccine, Low Dose. QIV-Split-HD: Quadrivalent Influenza Split-Virion Vaccine, Low Dose.
